# Global and regional assessments of the burden of transport injuries and associated risk factors, 1990–2021: Insights from the 2021 Global Burden of Disease study

**DOI:** 10.1097/MD.0000000000042157

**Published:** 2025-04-18

**Authors:** Shan-Hong Hu, Qin Wan

**Affiliations:** a Department of Emergency Medicine, Wenjiang People’s Hospital, Chengdu, China; b Department of Endocrinology and Metabolism, Affiliated Hospital of Southwest Medical University, Luzhou, China; c Metabolic Vascular Disease Key Laboratory of Sichuan Province, Luzhou, China; d Sichuan Clinical Research Center for Diabetes and Metabolism, Luzhou, China; e Sichuan Clinical Research Center for Nephropathy, Luzhou, China; f Cardiovascular and Metabolic Diseases Key Laboratory of Luzhou, Luzhou, China.

**Keywords:** age, Global Burden of Disease, risk factors, sex, transport injuries

## Abstract

The burden of transport injuries continues to constitute a major global challenge. This study seeks to utilize the most recent data from the 2021 Global Burden of Disease (GBD) study to comprehensively analyze the global and regional burden of transport injuries. This study utilized the most recent data from the 2021 GBD study to comprehensively analyze the trends in ASIRs, mortality rates, and disability-adjusted life years (DALYs) for transport injuries between 1990 and 2021. Subgroup analyses were performed based on gender, age, and geographic regions. Additionally, we examined potential risk factors associated with DALYs due to transport injuries. Between 1990 and 2021, the global burden of transport injuries exhibited an overall decreasing trend. Relative to 1990, the age-standardized incidence rate (ASIR) declined by approximately 37.1%, the age-standardized death rate (ASDR) decreased by approximately 33.3%, and the age-standardized DALY rate decreased by roughly 38.0%. Regional subgroup analysis demonstrated that regions with higher Social Development Index (SDI) exhibited higher ASIRs, but lower ASDRs and age-standardized DALY rates. At the same time, the converse was observed in regions with lower SDI. The burden of transport injuries decreased across all age cohorts. Furthermore, smoking, alcohol consumption, low bone mineral density, occupational injuries, and high temperatures were recognized as the 5 major risk factors for DALYs associated with transport injuries. This GBD 2021-based study provides further insights into the global burden of transport injuries from 1990 to 2021, with a particular focus on the impact of the COVID-19 pandemic. However, due to the inherent limitation of GBD studies, which lack rigorous harmonized standards, the quality of their data is highly dependent on individual country reports. As a result, the findings may deviate to some extent from actual conditions. In conclusion, although the global burden of transport injuries has generally declined, the disparities in transport injury burden across gender, age, and geographic regions warrant attention.

## 1. Introduction

Injuries represent a major cause of disability and mortality; however, they are often overlooked by healthcare professionals.^[1]^ Injury is defined as physical harm to the body resulting from unforeseen events or accidents.^[2]^ The International Classification of Diseases (ICD) currently classifies accidental injuries as a distinct category, encompassing traffic accidents, suffocation, drowning, electrocution, suicide, poisoning, and violence, as well as other physical, chemical, and biological factors.^[3]^ According to the 2019 Global Burden of Disease (GBD) study, road injuries were the leading cause of disability-adjusted life years (DALYs) attributable to injuries in 2019, ranking 7th, compared to 8th in 1990.^[4]^ As global urbanization progresses and the number of motor vehicles increases rapidly, the interactions between pedestrians, non vehicles, and motor vehicles have become more frequent, leading to varying levels of increased transport injury risk for all individuals.^[5]^

Although millions of disability and mortality incidents related to transport injuries occur worldwide each year, their impact varies significantly across age, gender, race, and socioeconomic status.^[6]^ For instance, previous studies have indicated that traffic accidents represent a more significant health threat in low- and middle-income countries, where approximately 90% of road traffic-related fatalities occur, leading to an economic loss of about 3% of GDP.^[7]^ The 2023 Global Road Safety Status Report published by the World Health Organization (WHO) indicates that although countries worldwide have implemented various laws and regulations to reduce the burden of both non and fatal traffic accidents, and the annual death toll from road traffic has decreased, the cost of transportation remains excessively high. Road transport injuries remain the leading cause of death among children and adolescents aged 5 to 29. Over half of these fatalities occur among pedestrians, cyclists, and motorcyclists, particularly in low- and middle-income countries.^[8]^ Considering that children often lack adequate road safety awareness and supervision from guardians, and that adolescents experience heightened emotional volatility during puberty, these factors may contribute to an increased burden of transport injuries in these age groups.^[9]^ This underscores the importance of analyzing the global age distribution of transport injury burden and tailoring intervention policies to specific age groups to more effectively prevent transport-related injuries.

Prior studies have primarily focused on the global burden of transport injuries before the 2019 COVID-19 pandemic; however, the burden of transport injuries during the pandemic remains substantial.^[10]^ The SARS-CoV-2 virus has led to a marked increase in the global burden of various diseases, overwhelming healthcare systems across the globe. In response to the burden imposed by COVID-19, governments and organizations worldwide have implemented stringent public health measures, including full or partial lockdowns, rigorous social distancing, restrictions on non travel, and prolonged closures of entertainment venues.^[11]^ One study found that during the full lockdown period, road traffic accidents resulting in minor or no injuries decreased significantly, while those leading to severe or fatal injuries did not exhibit a similar decline.^[12]^ These studies suggest that travel restrictions imposed during the COVID-19 pandemic may have significantly influenced the epidemiological distribution of transport injuries. However, it is important to note that the pandemic did not simply lead to a reduction in the global burden of transport injuries.

Accordingly, this study seeks to leverage the most recent data from GBD 2021 to investigate the evolving trends in the global epidemiology of transport injuries from 1990 to 2021, while addressing the data gap concerning changes in the related burden during the COVID-19 pandemic. Moreover, this study also aims to analyze the risk factors associated with transport injury-related DALYs, supporting relevant authorities in formulating more effective laws and regulations to achieve the global target of reducing road transport injuries and fatalities by 2030.

## 2. Method

### 2.1. Data source

The GBD 2021 study examined the epidemiology of over 370 diseases and injuries across 204 countries and regions worldwide, offering comprehensive data on incidence, mortality, and DALYs.^[13,14]^ All data were obtained from reputable public databases and meticulously screened to ensure data integrity.^[15]^ Mortality numbers and rates were primarily estimated using the cause-of-death ensemble model, whereas incidence numbers and rates were evaluated using Bayesian meta-regression models.^[16]^ Disability weight reflects the severity of health loss or nonfatal disability, whereas DALY represents the total years lost due to health impairment from the onset of disease to death.^[17]^ In summary, Years of Life Lost are calculated as the product of the number of deaths and the standard life expectancy at the age of death. In contrast, Years Lived with Disability (YLDs) are determined by multiplying the incidence rate of transport injuries by their respective disability weights. DALYs are then derived as the sum of years of life lost and YLDs.

### 2.2. Definition of transport injuries

In the GBD 2021 study, transport injuries are categorized into 2 groups: road injuries (including pedestrian, cyclist, motorcyclist, motor vehicle, and other road injuries) and other transport injuries. The ICD-10 codes used to define transport injuries are V00-V86.99, V87.2-V87.3, V88.2-V88.3, and V90-V98.8.

### 2.3. Data variables

The Socio-demographic Index (SDI) is a composite indicator that reflects a country development status, incorporating lagged per capita income distribution, the average education level of individuals aged 15 and above, and the total fertility rate for the population under 25. It is closely linked to health outcomes. SDI values range from 0 to 1, with 0 representing the lowest level of development and 1 representing the highest level.^[18]^ Based on SDI values, 204 countries and regions are classified into 5 categories: high SDI, medium-high SDI, medium SDI, low-medium SDI, and low SDI regions. Moreover, patients are divided into 5 age groups: under 5 years, 5 to 9 years, 10 to 19 years, 20 to 24 years, 25 to 29 years, 30 to 34 years, 35 to 39 years, 40 to 44 years, 45 to 49 years, 50 to 54 years, and 55 years and older.

### 2.4. Estimation of risk factor

The GBD 2021 includes 66 specific attributable risk factors, such as particulate matter pollution, high and low temperatures, lead exposure, smoking, secondhand smoke exposure, high red meat intake, high sodium intake, low fiber intake, low fruit and vegetable intake, and elevated fasting blood glucose, with comprehensive definitions of these factors provided in previous studies.^[19]^

### 2.5. Statistical analyses

Pearson correlation coefficients were employed to examine the potential association between changes in SDI values and the burden of transport injuries across global regions from 1990 to 2021. Statistical analysis was performed using GraphPad Prism (version 10.0.0 for Windows), including t-tests and ANOVA to compare differences in age-standardized incidence rates (ASIR), age-standardized mortality rates (ASDR), and age-standardized DALYs across different genders, age groups, and regions. These metrics take into consideration changes in the population age structure. Age standardization is employed to eliminate the effects of age distribution, thereby ensuring the comparability of research metrics across different populations. The GBD database estimates these indicators using the World Population Age Standard. All hypothesis tests were 2-tailed, with a significance level set at *P* < .05.

## 3. Result

### 3.1. Global trends and incidence of transport injuries

**Figure 1.**
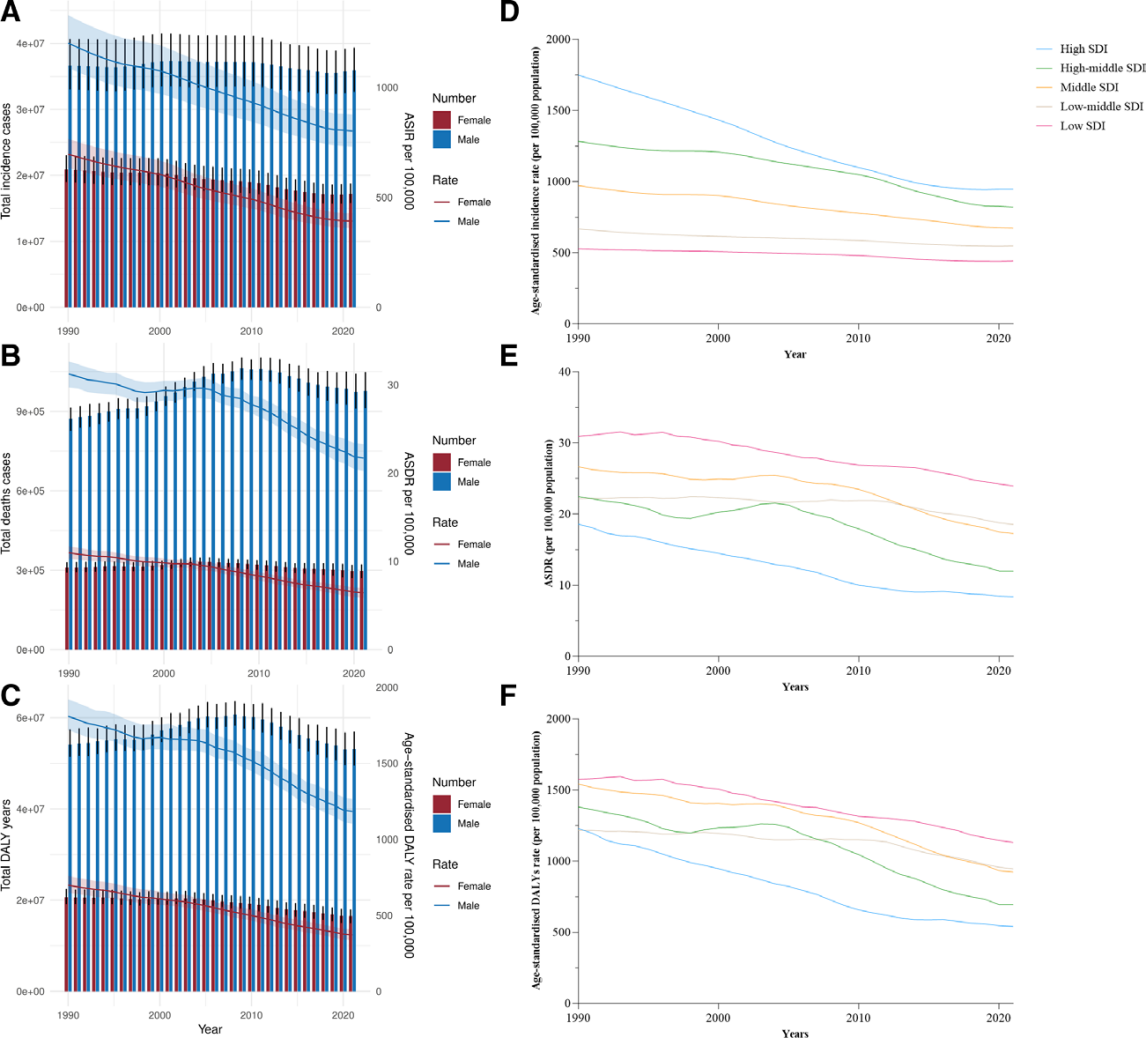
The change trends of traffic injury incidence cases, deaths cases and DALY years from 1990 to 2021. (A) The change trends of incidences. (B) The change trends of deaths. (C) The change trends of DALY. Blue bars represent males and orange bars represent females. (D) Trends from 1990 to 2021 in the ASIR of transport injuries in 5 SDI regions. (E) Trends from 1990 to 2021 in the ASDR of transport injuries in 5 SDI regions. (F) Trends from 1990 to 2021in the age-standardized DALY rate of transport injuries in 5 SDI regions. ASIR = age-standardized incidence rate, ASDR = age-standardized mortality rates, DALY = disability-adjusted life years, SDI = social development index.

As presented in Table 1, the global ASIR of transport injuries has demonstrated a consistent decline over the past 32 years, from 1055.73 cases per 100,000 in 1990 to 664.51 cases per 100,000 in 2021, reflecting a decrease of approximately 37.1%. The total number of transport injury cases decreased from 57,488,610.72 in 1990 to 57,488,610.72 in 2021. The total incidence and ASIR of transport injuries were consistently higher in males than in females. In 2021, the male ASIR for transport injuries was more than twice that of females (Fig. 1A). Furthermore, the ASIR of transport injuries in the 5 SDI regions exhibited varying degrees of decline. Notably, the ASIR of transport injuries in low SDI regions was the lowest, while in high SDI regions, it was the highest, at 527.41/100,000 and 946.52/100,000, respectively (Fig. 1D). Regionally, except in South Asia, the ASIR of transport injuries decreased in all other 12 regions. The ASIR of South Asia increased slightly from 408.57/100,000 in 1990 to 410.62/100,000 in 2021, with the absolute number of cases rising from 4,473,266.60 to 7,721,386.28, reflecting an increase of 72.6%. In contrast, the ASIR in Australasia and Western Europe decreased by more than 50%.

**Table 1:** The incidence rate of transport injuries in 1990/2021.

	1990 cases	1990 rate	2021 cases	2021 rate
Overall	57,488,610.72	1055.73	53,089,007.65	664.51
Sex				
Male	36,606,001.70	1335.31	35,910,905.67	891.10
Female	20,882,609.03	772.62	17,178,101.98	435.80
SDI				
High SDI	15,299,069.27	1749.34	9,896,808.52	946.52
High-middle SDI	13,948,594.22	1283.07	10,864,552.63	820.99
Middle SDI	17,365,753.51	972.30	16,956,369.98	672.94
Low-middle SDI	8,000,997.81	666.97	10,579,603.20	548.66
Low SDI	2,819,576.14	527.41	4,752,313.14	441.98
Region				
Australasia	356,717.94	1743.25	210,718.63	717.13
Central Asia	570,608.27	796.77	579,500.45	589.64
Central Europe	1,662,470.14	1356.65	734,658.12	685.59
East Asia	12,547,214.33	1001.00	12,736,478.92	820.24
Eastern Europe	3,870,320.23	1740.65	1,984,040.28	1040.46
Latin America and Caribbean	4,276,456.24	1070.60	4,558,575.86	746.84
North Africa and Middle East	6,680,957.65	1823.63	7,180,905.47	1136.00
Oceania	45,309.00	691.71	87,079.10	619.71
South Asia	4,473,266.60	408.57	7,721,386.28	410.62
Southeast Asia	4,135,159.75	846.09	3,588,905.41	499.83
Southern Latin America	523,456.15	1052.64	659,497.25	967.10
Sub-Saharan Africa	2,944,274.16	585.12	4,624,373.47	434.20
Western Europe	6,260,414.80	1662.05	2,622,053.10	660.88

SDI = social development index.

### 3.2. Global trends and death of transport injuries

As presented in Table 2, over the past 32 years, the global ASDR for transport injuries has generally decreased, from 23.29 cases per 100,000 in 1990 to 15.54 cases per 100,000 in 2021, reflecting a reduction of approximately 33.3%. The absolute number of transport injury deaths increased from 1,180,563.69 in 1990 to 1,274,143.19 in 2021. The total incidence and ASIR of transport injuries in males have consistently exceeded those in females. In 2021, the male ASDR for transport injuries was more than 3 times that of females. Although the ASDR for both males and females decreased, the absolute number of male deaths increased (Fig. 1B). Additionally, the ASDRs for transport injuries in the 5 SDI regions generally exhibited varying degrees of decline. The highest ASDR was observed in low SDI regions, while the lowest was observed in high SDI regions, with ASDRs of 23.96/100,000 and 8.36/100,000, respectively (Fig. 1E). Regionally, the ASDR for transport injuries declined in all 13 regions, with the most significant decline occurring in Western Europe, at 76.0%.

**Table 2:** The death of transport injuries in 1990/2021.

	1990 cases	1990 rate	2021 cases	2021 rate
Overall	1,180,563.69	23.29	1,274,143.19	15.54
Sex				
Male	871,206.42	34.69	977,360.36	24.10
Female	309,357.26	12.18	296,782.83	7.14
SDI				
High SDI	174,308.08	18.57	108,950.73	8.36
High-middle SDI	240,487.27	22.44	183,433.32	11.98
Middle SDI	419,609.98	26.66	451,067.99	17.27
Low-middle SDI	218,474.84	22.12	330,628.34	18.54
Low SDI	126,533.09	30.94	199,138.54	23.96
Region				
Australasia	3861.31	18.17	2008.95	5.71
Central Asia	14,722.26	23.18	10,319.06	10.74
Central Europe	28,460.59	21.56	11,353.36	8.04
East Asia	261,213.85	22.49	261,761.48	14.99
Eastern Europe	66,227.76	27.82	25,931.00	11.32
Latin America and Caribbean	96,646.27	28.56	103,990.04	16.64
North Africa and Middle East	113,425.29	37.90	121,336.83	20.75
Oceania	1221.93	22.33	2314.13	18.63
South Asia	165,298.11	18.18	299,354.94	16.92
Southeast Asia	119,170.14	28.48	132,222.72	18.62
Southern Latin America	7430.66	15.41	7986.24	10.70
Sub-Saharan Africa	141,791.98	36.22	215,830.08	26.55
Western Europe	70,046.58	16.51	22,197.74	3.96

SDI = social development index.

### 3.3. Global trends and DALY of transport injuries

As presented in Table 3, the global age-standardized DALY rate for transport injuries exhibited a consistent downward trend over the past 32 years, declining from 1395.51 years per 100,000 in 1990 to 864.63 years per 100,000 in 2021, representing a reduction of approximately 38.0%. The absolute DALY years attributable to transport injuries decreased from 74,775,334.38 years in 1990 to 69,629,520.26 years in 2021. The DALY years and age-standardized DALY rates among males consistently exceeded those among females. In 2021, the age-standardized DALY rate for males was more than 3 times that for females (Fig. 1C). Similarly, across the 5 SDI regions, the age-standardized DALY rates for transport injuries showed varying extents of decline. Among the 5 SDI regions, low SDI regions recorded the highest age-standardized DALY rate, whereas high SDI regions registered the lowest, at 1132.85 per 100,000 and 540.22 per 100,000, respectively (Fig. 1F). At the regional level, all 13 areas reported reductions in the age-standardized DALY rate for transport injuries, with Western Europe achieving the most substantial decline, at 74.3%.

**Table 3:** The DALY of transport injuries in 1990/2021.

	1990 yr	1990 rate	2021 yr	2021 rate
Overall	74,775,334.38	1395.51	69,629,520.26	864.63
Sex				
Male	54,117,310.22	2009.52	53,151,578.76	1314.08
Female	20,658,024.16	776.52	16,477,941.50	411.95
SDI				
High SDI	11,173,867.47	1228.81	6,441,730.71	540.22
High-middle SDI	14,963,281.46	1380.35	9,859,513.38	693.67
Middle SDI	26,679,465.95	1542.33	23,724,184.75	923.82
Low-middle SDI	13,790,663.07	1217.48	18,017,167.77	945.99
Low SDI	8,097,364.04	1575.03	11,534,598.18	1132.85
Region				
Australasia	259,585.67	1241.28	127,879.74	390.23
Central Asia	898,267.72	1325.13	605,632.45	617.09
Central Europe	1,659,330.99	1296.37	609,133.38	486.30
East Asia	16,737,628.20	1371.36	13,301,917.91	822.43
Eastern Europe	4,021,898.72	1738.63	1,585,828.15	731.90
Latin America and Caribbean	5,983,121.33	1584.90	5,790,475.67	931.49
North Africa and Middle East	7,788,631.73	2263.72	7,082,600.84	1136.72
Oceania	77,685.31	1233.32	144,232.19	1045.98
South Asia	9,862,606.63	950.74	15,558,042.91	829.27
Southeast Asia	7,559,627.62	1610.33	7,054,915.64	977.37
Southern Latin America	447,465.20	913.01	480,279.70	667.08
Sub-Saharan Africa	9,006,463.52	1823.91	12,420,270.93	1223.93
Western Europe	4,452,794.65	1109.33	1,395,665.81	284.98

SDI = social development index.

### 3.4. Association between SDI and global incidence, death and DALY rates of transport injuries

**Figure 2.**
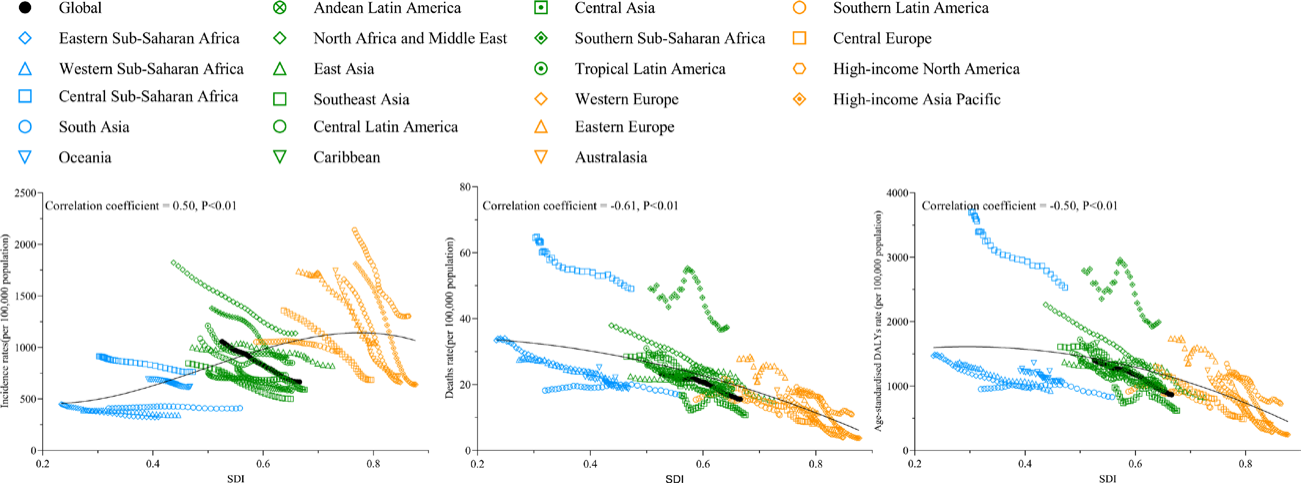
The change trends and correlation analyses of ASIR, ASDR and age-standardized DALY rate of transport injuries with SDI from 1990 to 2021. ASIR = age-standardized incidence rate, ASDR = age-standardized mortality rates, DALY = disability-adjusted life years, SDI = social development index.

The relationship between SDI trends and ASIR, ASDR, and age-standardized DALY rates was evaluated across 21 global regions from 1990 to 2021. Pearson correlation analysis results indicated a significant positive correlation between transport injuries ASIR and SDI at both global and regional levels (correlation coefficient = 0.50, *P* < .01). Conversely, transport injuries-related ASDR and age-standardized DALY rates showed significant negative correlations with SDI, with correlation coefficients of −0.61 and −0.50, respectively (*P* < .01) (Fig. 2).

### 3.5. Age distribution of transport injuries DALY

**Figure 3.**
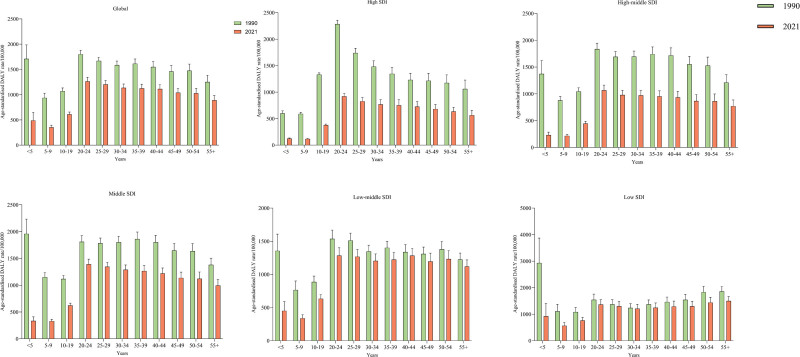
The age-standardized DALY rate of transport injuries in different age groups from 1990 to 2021. DALY = disability-adjusted life years.

Age-standardized DALY rates associated with transport injuries were analyzed across 11 age groups globally and within 5 SDI regions spanning the period from 1990 to 2021. The analysis indicated a decline in age-standardized DALY rates related to transport injuries across all age groups compared to 1990, with the most significant reduction observed in children under 5 years of age. Notably, individuals aged 20 to 24 years in high SDI regions exhibited the highest age-standardized DALY rates attributable to transport injuries (Fig. 3).

### 3.6. Risk factors contributing to transport injuries-related DALY

**Figure 4.**
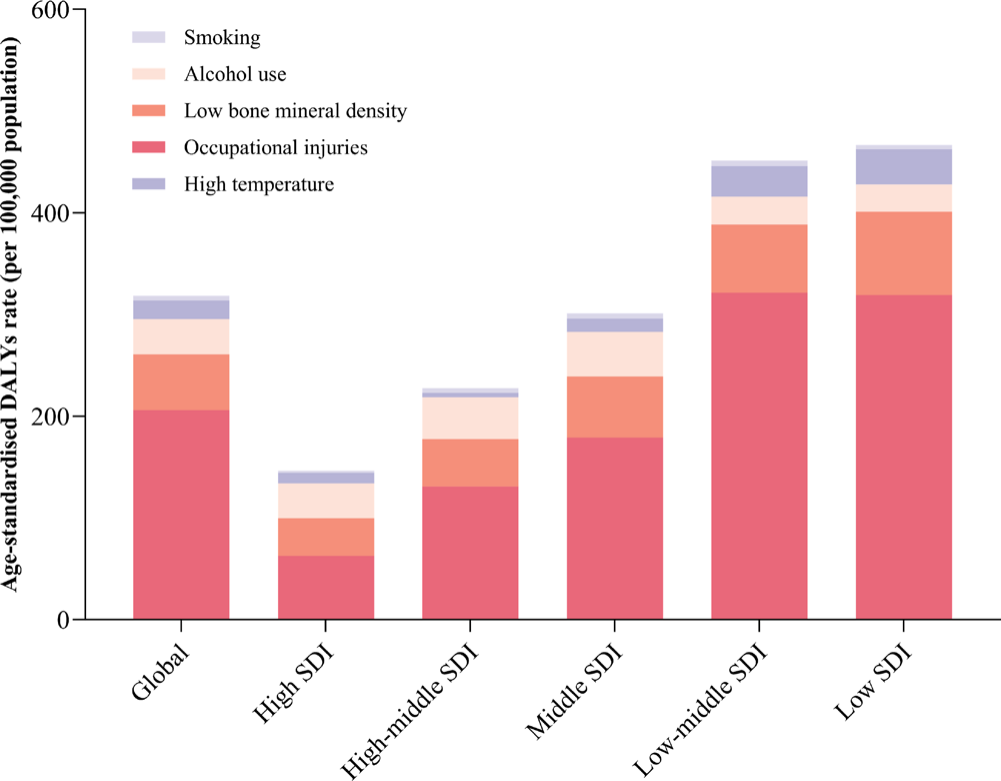
Risk factors contributing to transport injuries-related DALY. DALY = disability-adjusted life years.

Additionally, 5 key risk factors contributing to transport injuries-related DALYs were identified: smoking, alcohol consumption, low bone mineral density, occupational hazards, and high temperature. Among these factors, occupational hazards accounted for the highest proportion of transport injuries-related DALYs globally and across all regions, whereas smoking made the smallest contribution (Fig. 4).

## 4. Discussion

This study utilized the latest GBD 2021 data to conduct a comprehensive analysis of the ASIR, ASDR, and age-standardized DALYs for transport injuries, stratified by gender, SDI levels, and age groups, at both global and regional levels from 1990 to 2021. In addition, the study identified key risk factors contributing to DALYs related to transport injuries. Firstly, the findings indicate that the global burden of transport injuries has significantly decreased over the past 3 decades, reflecting substantial progress made by countries worldwide in addressing this issue. However, the disease burden varies markedly across regions and is closely linked to differences in social and cultural factors. Gradient differences in SDI significantly exacerbate disparities in the burden of transport injuries. High SDI regions exhibited the highest ASIR and the lowest ASDR and age-standardized DALY rates for transport injuries, whereas the opposite was true for low SDI regions. The burden of transport injuries was found to be lowest in North Africa and the Middle East. Thirdly, men continue to bear the greatest burden of transport injuries. Fourthly, smoking, alcohol consumption, low bone mineral density, occupational hazards, and high temperature are the 5 primary factors contributing to DALYs associated with transport injuries. Therefore, given the still substantial burden of transport injuries, there is an urgent need for more targeted and effective interventions.

In terms of the time span, even during the COVID-19 pandemic, the global burden of transport injuries generally exhibited a decreasing trend, which is largely consistent with existing studies. The COVID-19 pandemic did not appear to have a significant impact on the overall epidemiological trend of transport injuries. However, considering regional policy differences during the pandemic, the available data may not be entirely comprehensive, and further detailed investigations are needed to explore these trends more thoroughly in the future. In this study, the burden of transport injuries in males was significantly higher than in females, which aligns with previous research findings.^[20]^ This is likely because males tend to drive more frequently and engage in high-risk behaviors, such as speeding, reckless driving, and improper use of safety equipment.^[21]^ Alcohol consumption and smoking are critical risk factors contributing to the elevated burden of transport injuries in males, as males are more prone than females to engage in these behaviors.^[22]^ A study examining child transport injuries found that boys (11.5 times) travel more frequently on the road each day compared to girls (9.6 times).^[23]^ Moreover, boys may be more inclined to make poor decisions in risky situations, while girls’ more cautious tendencies may mitigate their exposure to risk.^[24]^

Notably, the ASIR and ASDR for transport injuries exhibit contrasting epidemiological trends across different SDI regions. In high SDI regions, the transport injuries ASIR is the highest, while the ASDR and age-standardized DALY rates are the lowest; conversely, low SDI regions display the opposite trend. Specifically, although the transport injuries ASIR in Europe have shown a declining trend, accidental injuries remain the second leading cause of death among individuals aged 5 to 29 years.^[25]^ Additionally, in the early 21st century, Russia had the highest number of road traffic accidents globally, with traffic mortality rates twice those of other G-8 countries.^[26,27]^ Consequently, countries worldwide began implementing various laws and regulations to enhance road safety, including banning alcohol consumption for drivers, mandating seat belt use for both drivers and passengers and improving traffic signals.^[28,29]^ Moreover, organizations such as the Global Road Safety Partnership have played a pivotal role in providing funding and technical support for transportation safety.^[30]^ The relative decline in ASDR and age-standardized DALY rates in countries with a high SDI can be attributed to advancements in medical technology and healthcare resources. The superior accessibility of healthcare in these regions enables more effective treatment of patients with transport injuries, significantly reducing the risk of disability and mortality.

In general, low SDI and lower-middle SDI regions continue to bear the greatest share of the transport injury burden. This is likely attributed to inadequate healthcare measures for transport injuries, insufficient road safety awareness, substandard road conditions, and low political investment in these regions.^[31]^ For example, the rapid economic growth of certain cities has resulted in a significant increase in the number of motor vehicles. However, frequent traffic accidents occur due to a lack of road safety awareness and preventive measures.^[32]^ Additionally, the characteristics of motorcycles, such as high speed, instability, and inadequate safety features, contribute to alarmingly high traffic accidents and mortality rates in regions where motorcycles are predominantly used for transport.^[33]^ Furthermore, as the global population continues to grow and urbanization intensifies, road transportation systems face immense pressure in densely populated countries.^[34]^

In terms of age, our findings indicate that the burden of transport injuries was highest in the 20 to 24 age group, particularly in high SDI regions in 1990. Fortunately, the implementation of numerous laws and regulations in recent years, including the driving license system, minimum drinking age laws, seatbelt and helmet usage requirements, and speed limit enforcement near schools, residential areas, and recreational zones, has significantly reduced the transport injury burden for this age group.^[35]^ Regarding risk factors, occupational injuries were the most significant, likely due to the increased likelihood of drivers or other road workers encountering various vehicles and pedestrians, thus elevating the risk of transport injuries.^[36]^ Therefore, enhancing safety education and improving protective measures for these transport workers represent critical public health priorities. Moreover, the DALY associated with transport injuries due to low bone mineral density should not be overlooked, as fractures are the most immediate form of injury resulting from transportation accidents.^[37]^ Notably, we observed the most significant decline in age-standardized DALY rates among infants under 5 years of age compared to 1990. This decline may be attributed to economic development, which has facilitated the widespread adoption of child road safety measures, such as vehicle child seats.^[38]^

This study presents several notable advantages. Firstly, by leveraging the high-quality evidence and methodological framework provided by the GBD 2021, we have accurately depicted the global and regional burden of transport injuries spanning from 1990 to 2021. Secondly, in contrast to existing studies, our research primarily provides an updated time frame, ensuring the timeliness and relevance of the findings. Nevertheless, this study does have several limitations. The results are primarily based on aggregated data from the GBD study, and the accuracy of these data depends on the quality of reporting across various countries, given that no universally accepted reporting standard exists. Furthermore, certain countries and regions may exhibit a high number of unreported transport injury cases, and insufficient data on associated risk factors could compromise the accuracy of the study’s results. Of course, in future studies, we will build upon the findings of the GBD study and integrate reliable data from other official organizations to enhance the robustness and reliability of our results. Second, the version of the ICD used to assess mortality transitioned from ICD-9 to ICD-10 during the study period, and this evolution in classification could have influenced the results. Second, the version of the ICD used to assess mortality transitioned from ICD-9 to ICD-10 during the study period, and this evolution in classification could have influenced the results. Fifth, since incidence rates and DALYs are closely linked to morbidity, disease duration, and survival, they are influenced by the treatment options and capabilities available in each region. As noted previously, high-income regions may also exhibit higher DALYs due to their greater access to treatment and the higher quality of care provided. Moreover, treatment methods are not only influenced by the overall economic level of the region but also by the experience of local medical staff and the doctor-patient relationship within society, with these differences in medical conditions potentially having a significant impact on disease prognosis.

## 5. Conclusions

In conclusion, despite the overall decline in the global burden of transport injuries over the 32 years from 1990 to 2021, significant disparities in burden across gender, age, and region persist, particularly among men, with the highest ASIR for transport injuries in high SDI regions and the highest ASDR and DALY in low SDI regions. Smoking, alcohol consumption, low bone mineral density, occupational hazards, and high temperature constitute the 5 principal risk factors contributing to transport injuries. Therefore, relevant public health organizations should formulate more cost-effective and targeted strategies – such as supporting developing countries in improving road and medical infrastructure and strengthening road safety education for children and adolescents – based on the distributional characteristics of the burden of transport injuries. These efforts are essential to achieving the WHO’s Sustainable Development Goal of halving the global number of deaths and injuries caused by road traffic crashes by 2030.

## Acknowledgments

We extend our sincere gratitude to the GBD team for granting us access to their extensive database.

## Author contributions

**Data curation:** Shan-Hong Hu.

**Funding acquisition:** Qin Wan.

**Methodology:** Shan-Hong Hu.

**Validation:** Qin Wan.

**Visualization:** Shan-Hong Hu.

**Writing – original draft:** Shan-Hong Hu.

**Writing – review & editing:** Qin Wan.
